# Three new acid *M* ^+^ arsenates and phosphates with multiply protonated As/PO_4_ groups

**DOI:** 10.1107/S2053229619008489

**Published:** 2019-07-25

**Authors:** Karolina Schwendtner, Uwe Kolitsch

**Affiliations:** a Institute for Chemical Technology and Analytics, Division of Structural Chemistry, TU Wien, Getreidemarkt 9, Wien 1060, Austria; b Mineralogisch-Petrographische Abteilung, Naturhistorisches Museum, Burgring 7, Wien 1010, Austria; c Institut für Mineralogie und Kristallographie, Universität Wien, Althanstrasse 14, Wien 1090, Austria

**Keywords:** arsenate, phosphate, caesium, lithium, ammonium, statistical evaluation, crystal structure, H_2_PO_4_, H_2_AsO_4_, H_3_AsO_4_, As—O bond lengths

## Abstract

The crystal structures of caesium di­hydrogen arsenate(V) bis­[tri­hydrogen arsen­ate(V)], Cs(H_2_AsO_4_)(H_3_AsO_4_)_2_, ammonium di­hydrogen arsenate(V) tri­hydrogen arsenate(V), NH_4_(H_2_AsO_4_)(H_3_AsO_4_), and dilithium bis­(di­hydrogen phosphate), Li_2_(H_2_PO_4_)_2_, were solved from single-crystal X-ray diffraction data. NH_4_(H_2_AsO_4_)(H_3_AsO_4_), which was hydro­thermally synthesized (*T* = 493 K), is homeotypic with Rb(H_2_AsO_4_)(H_3_AsO_4_), while Cs(H_2_AsO_4_)(H_3_AsO_4_)_2_ crystallizes in a novel structure type and Li_2_(H_2_PO_4_)_2_ represents a new polymorph of this composition. The Cs and Li compounds grew at room temperature from highly acidic aqueous solutions. Li_2_(H_2_PO_4_)_2_ forms a three-dimensional (3D) framework of PO_4_ tetra­hedra sharing corners with Li_2_O_6_ dimers built of edge-sharing LiO_4_ groups, which is reinforced by hydrogen bonds. The two arsenate compounds are characterized by a 3D network of AsO_4_ groups that are connected solely *via* multiple strong hydrogen bonds. A statistical evaluation of the As—O bond lengths in singly, doubly and triply protonated AsO_4_ groups gave average values of 1.70 (2) Å for 199 As—OH bonds, 1.728 (19) Å for As—OH bonds in HAsO_4_ groups, 1.714 (12) Å for As—OH bonds in H_2_AsO_4_ groups and 1.694 (16) Å for As—OH bonds in H_3_AsO_4_ groups, and a grand mean value of 1.667 (18) Å for As—O bonds to nonprotonated O atoms.

## Abstract 2

The crystal structures of Cs(H_2_AsO_4_)(H_3_AsO_4_)_2_, NH_4_(H_2_AsO_4_)(H_3_AsO_4_) and Li_2_(H_2_PO_4_)_2_ were determined from single-crystal X-ray diffraction data. The two alkali compounds represent novel structure types, while the ammonium compound is homeotypic with its Rb analogue.

## Introduction   

*M* ^+^ phosphates and arsenates, and their crystal structures and physicochemical properties, have been extensively studied. Several compounds exhibit inter­esting properties, such as protonic conductivity (Chouchene *et al.*, 2017*a* ▸,*b* ▸; Volkov *et al.*, 1995 ▸, 1997 ▸; Voronov *et al.*, 2013 ▸; Dekhili *et al.*, 2018 ▸) or nonlinear optical properties (Dhouib *et al.*, 2014*a* ▸, 2017 ▸; Kumaresan *et al.*, 2008 ▸).

To further increase the knowledge about the possible compounds and structure types of *M* ^+^–*M* ^3+^ arsenates, a comprehensive study of the system *M* ^+^–*M* ^3+^–O–(H–)As/P^5+^ (*M* ^+^ = Li, Na, K, Rb, Cs, Ag, Tl and NH_4_; *M* ^3+^ = Al, Ga, In, Sc, Fe, Cr and Tl) was undertaken, which led to a large number of new structure types that have been published (Schwendtner, 2006 ▸; Schwendtner & Kolitsch, 2004*a* ▸,*b* ▸, 2005 ▸, 2007*a* ▸,*b* ▸,*c* ▸, 2017*a* ▸,*b* ▸, 2018 ▸). The three compounds structurally characterized in the present article are by-products of this comprehensive study. The following paragraphs provide brief backgrounds to the families of materials to which the three compounds belong.

Lithium phosphates are rather common and the system Li–H–P–O has been widely studied because of the proton conductivity of compounds like LiH_2_PO_4_ (Catti & Ivaldi, 1978 ▸). The title compound Li_2_(H_2_PO_4_)_2_ is a new polymorph of this well-known compound. Other known compounds in the Li–H–P–O system, the majority containing polymerized phos­phate groups, include Li_4_H(PO_3_)_5_, LiH_2_PO_2_, Li_6_(P_6_O_18_)(H_2_O)_3_, Li_4_P_2_O_8_(H_2_O)_4_, Li_3_(P_3_O_9_)(H_2_O)_3_, Li_6_(P_6_O_18_)(H_2_O)_5_, Li_4_(P_4_O_12_)(H_2_O)_5_, Li_6_(P_6_O_18_)(H_2_O)_8.24_, Li_6_(P_6_O_18_)(H_2_O)_9.86_, Li_3_PO_4_ and Li_4_P_2_O_7_.

Known caesium arsenates include CsAs_3_O_8_ (Schwendtner & Kolitsch, 2007*a* ▸), Cs_3_AsO_4_ (Emmerling *et al.*, 2002 ▸), Cs_2_(HAsO_4_)(H_2_O)_2_ (Stöger & Weil, 2014 ▸), KDP-type Cs(H_2_AsO_4_) (Ferrari *et al.*, 1956 ▸) and CsH_5_(AsO_4_)_2_ (Naili *et al.*, 2001 ▸). Ammonium arsenate compounds comprise (NH_4_)(H_2_AsO_4_), for which a tetra­gonal KDP-type polymorph (Khan & Baur, 1972 ▸) and an ortho­rhom­bic low-temperature polymorph (Fukami, 1989 ▸) were reported, (NH_4_)_2_(HAsO_4_) (Weil, 2012 ▸) and (NH_4_)_3_(AsO_4_)(H_2_O)_3_ (Hseu & Lu, 1977 ▸).

Compounds containing H_3_AsO_4_ groups are relatively rare and mainly known from compounds containing organic groups (*e.g.* Dekola *et al.*, 2011 ▸; Dhouib *et al.*, 2014*a* ▸,*b* ▸, 2017 ▸; Ratajczak *et al.*, 2000 ▸). Inorganic compounds containing arsenic acid (with clearly located H atoms of the H_3_AsO_4_ group) and with known crystal structures are restricted to only seven representatives: CuH_10_(AsO_4_)_4_ (Tran Qui & Chiadmi, 1986 ▸) and isotypic ZnH_10_(AsO_4_)_4_ (Sure & Guse, 1989 ▸) (the O—H bonds were not clearly identified in the latter structure determination), RbH_5_(AsO_4_)_2_ (Naili & Mhiri, 2001 ▸), CsH_5_(AsO_4_)_2_ (Naili *et al.*, 2001 ▸), K_4_(SO_4_)(HSO_4_)_2_(H_3_AsO_4_) (Amri *et al.*, 2007 ▸), Cs_4_(SeO_4_)(HSeO_4_)_2_(H_3_AsO_4_) (Amri *et al.*, 2009 ▸) and isotypic Rb_4_(SO_4_)(HSO_4_)_2_(H_3_AsO_4_) (Belhaj Salah *et al.*, 2018 ▸). (NH_4_)_2_(H_3_AsO_4_)(SO_4_) (Boubia *et al.*, 1985 ▸) also con­tains H_3_AsO_4_ groups, but the H atoms were not located, and for CdH_10_(AsO_4_)_4_ (Tran Qui & Chiadmi, 1986 ▸), hydro­gen-bond details were published, but no atomic coordinates.

## Experimental   

### Synthesis and crystallization   

Analytical grade chemicals were used for all syntheses. NH_4_(H_2_AsO_4_)(H_3_AsO_4_) was grown by hydro­thermal methods (*T* = 493 K, 7 d, Teflon-lined stainless steel autoclave) from a mixture of In_2_O_3_ and H_3_AsO_4_·0.5H_2_O in an approximate volume ratio of 1:10 and 10 drops of NH_4_(OH) (32%). No additional H_2_O was added. The reaction product was a solid mass of colourless inter­grown crystals with less than 10 vol% of a yellow unidentified material. The NH_4_(H_2_AsO_4_)(H_3_AsO_4_) crystals are stable in air.

Cs(H_2_AsO_4_)(H_3_AsO_4_)_2_ formed as the secondary product from further reaction of hydrothermally grown CsAs_3_O_8_ (Schwendtner & Kolitsch, 2007*a* ▸). CsAs_3_O_8_ contains AsO_6_ groups, is highly hygroscopic and, at room temperature, decomposes to a highly acidic liquid in which rounded prismatic glassy colourless crystals of Cs(H_2_AsO_4_)(H_3_AsO_4_)_2_ grew within a few weeks.

Li_2_(H_2_PO_4_)_2_ was also a secondary product of a hydro­thermal run (*T* = 493 K, 7 d, Teflon-lined stainless steel auto­clave) from a mixture of Li_2_CO_3_, Ga_2_O_3_, phospho­ric acid and distilled water. The initial and final pH values were both about 1. The hydro­thermal synthesis yielded globular crystal aggregates of rounded hexa­gonal prisms of GaPO_4_. From the remaining acidic liquid of the synthesis, Li_2_(H_2_PO_4_)_2_ grew as colourless crude block-shaped crystals by slow evaporation at room temperature.

### Refinement   

Crystal data, data collection and structure refinement details are summarized in Table 1 ▸. NH_4_(H_2_AsO_4_)(H_3_AsO_4_) disintegrated (‘melted’) during the measurement, so only the first two sets or 65% of the Ewald sphere could be measured. Specifically, we note that out of the nine sets collected, the first two were fully usable (no decay visible); the decay only started with set 3, so we ignored sets 3–9. We did not observe any anomalous behaviour of the data set during scaling. The remaining sets showed a pseudocubic *I*-centred tetra­gonal unit cell, with approximate *a* and *c* values of 7.68 and 7.69 Å, respectively; possibly NH_4_(H_2_AsO_4_)(H_3_AsO_4_) recrystallized to pseudocubic *I* 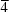
2*d*-type (NH_4_)H_2_AsO_4_ (Fukami, 1989 ▸). Nine reflections with negative intensities (blocked by the beam stop) were omitted from the refinement. All N—H and O—H bonds were restricted to 0.9 ± 0.2 Å, as was the O6—H6 bond in Cs(H_2_AsO_4_)(H_3_AsO_4_)_2_. The O—H bonds in Li_2_(H_2_PO_4_)_2_ were not restrained as they refined to reasonable values for refinements based on the X-ray diffraction data sets.

## Results and discussion   

**Figure 1:**
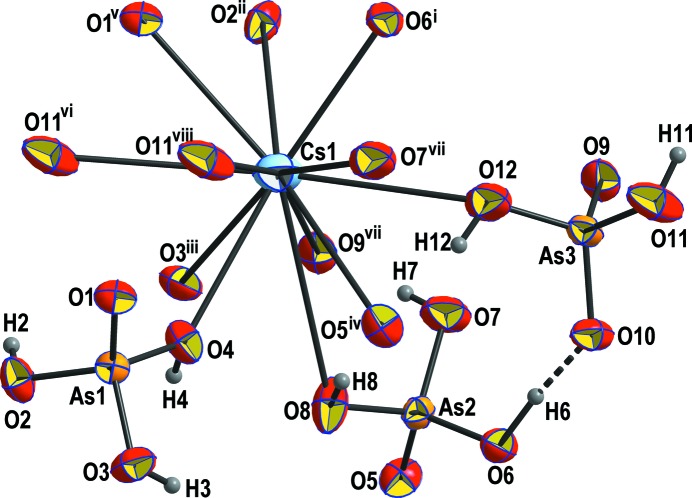
The principal building unit of Cs(H_2_AsO_4_)(H_3_AsO_4_)_2_, shown as dis­placement ellipsoids at the 70% probability level. The symmetry codes are as defined in Table 2 ▸.

**Figure 2:**
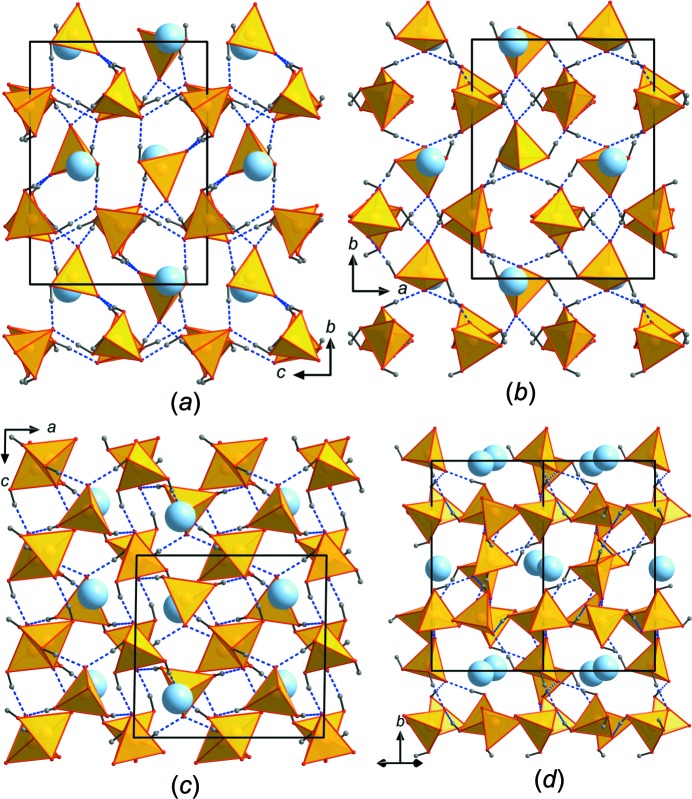
Structural drawings of novel Cs(H_2_AsO_4_)(H_3_AsO_4_)_2_, viewed along (*a*) *a*, (*b*) *c*, (*c*) *b* and (*d*) [101]. The unit cell is outlined. AsO_4_ tetra­hedra (yellow) are connected *via* multiple hydrogen bonds (blue) into a 3D network. The Cs^+^ cations lie between the AsO_4_ tetra­hedra.

The asymmetric unit of Cs(H_2_AsO_4_)(H_3_AsO_4_)_2_ contains one Cs, three As, 12 O and eight H atoms (Fig. 1 ▸). The Cs atom is 12-coordinated, with the Cs—O bond lengths varying between 3.1202 (17) and 3.934 (3) Å (Table 2 ▸). The average Cs—O bond length (3.458 Å) is considerably longer than the statistical average of 3.377 Å for 12-coordinated Cs atoms (Gagné & Hawthorne, 2016 ▸), explaining the low bond-valence sum (BVS; Gagné & Hawthorne, 2015 ▸) of 0.85 v.u. The As—O bond lengths are very similar for the doubly (As3) and triply protonated (As1 and As2) As atoms (1.683–1.681 Å) and slightly shorter than the statistical average of 1.687 Å (Gagné & Hawthorne, 2018*a* ▸). Since two/three O atoms of the coordination polyhedra are protonated, the As—O bond lengths are only slightly elongated compared to unprotonated O atoms. The BVSs of the three As atoms are between 5.06 and 5.09 v.u. and thus close to the expected value, whereas all O atoms are considerably underbonded, with BVSs ranging from 1.22 to 1.53 v.u., and are all either donors or acceptors of hydrogen bonds. The latter are strong (compared to the other H_3_AsO_4_-containing compounds cited above), with O—H⋯O distances in the range 2.524 (2)–2.664 (2) Å (Table 3 ▸) and connect the individual protonated AsO_4_ tetra­hedra into a three-dimensional (3D) network (Figs. 2 ▸ *a*–*c*). In the [101] direction, the structure forms tunnels walled by AsO_4_ tetra­hedra in which the Cs atom is located (Fig. 2 ▸ *d*).

**Figure 3:**
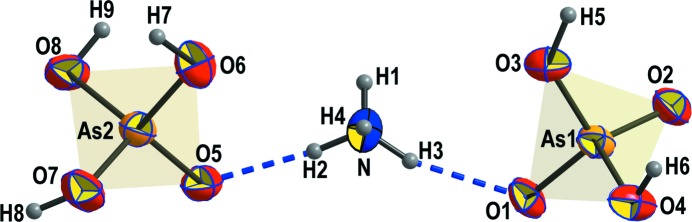
The principal building unit of (NH_4_)(H_2_AsO_4_)(H_3_AsO_4_), shown as dis­placement ellipsoids at the 70% probability level. Hydrogen bonds are shown as blue dashed lines.

**Figure 4:**
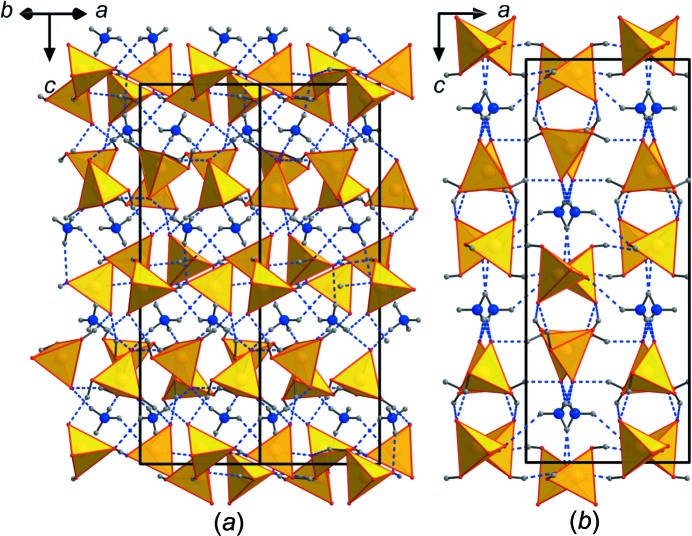
Structural drawings of (NH_4_)(H_2_AsO_4_)(H_3_AsO_4_), viewed along (*a*) [110] and (*b*) *b*. The unit cell is outlined. AsO_4_ tetra­hedra (yellow) are connected *via* multiple hydrogen bonds (blue dashed lines) into a 3D network. AsO_4_ tetra­hedra and NH_4_^+^ cations are arranged in layers perpendicular to *c*. Additional hydrogen bonds of medium strength are provided by the NH_4_^+^ cations.

**Figure 5:**
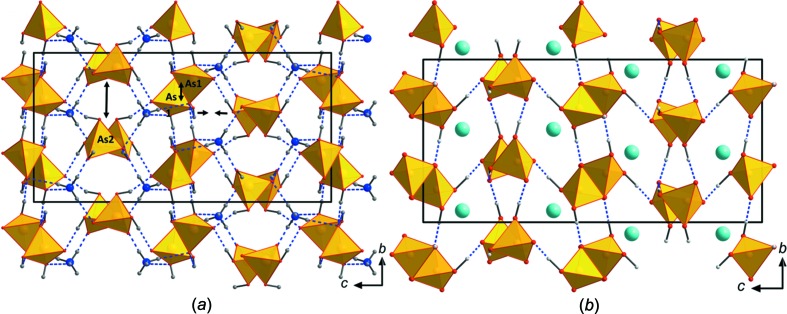
Comparison of (*a*) homeotypic (NH_4_)(H_2_AsO_4_)(H_3_AsO_4_) with (*b*) Rb(H_2_AsO_4_)(H_3_AsO_4_) (Naili & Mhiri, 2001 ▸). A shift (arrows in figure) of As1 in the *b* direction leads to a compression of the whole structure along *c*, and results in a change of the hydrogen-bonding network. Hydrogen bonds connecting As2O_4_ tetra­hedra along *b* are lost in (NH_4_)(H_2_AsO_4_)(H_3_AsO_4_), but new hydrogen bonds now connect As1O_4_ and As2O_4_ along *c*.

The structure of (NH_4_)(H_2_AsO_4_)(H_3_AsO_4_) is homeotypic with that of Rb(H_2_AsO_4_)(H_3_AsO_4_) (Naili & Mhiri, 2001 ▸); the Rb^+^ cation is replaced by an NH_4_^+^ group providing additional hydrogen bonds to the atomic arrangement. This structure type is also closely related to that of CsH_5_(AsO_4_)_2_ (Naili *et al.*, 2001 ▸), which can be seen as a distorted version of the Rb(H_2_AsO_4_)(H_3_AsO_4_) structure type. The structure of (NH_4_)(H_2_AsO_4_)(H_3_AsO_4_) is built of individual, doubly or triply protonated AsO_4_ tetra­hedra that are connected *via* strong hydrogen bonds into a 3D network (Figs. 3 ▸, 4 ▸ *a* and 4*b*). The NH_4_^+^ groups lie in voids and further reinforce the network *via* medium-to-weak strength hydrogen bonds. AsO_4_ tetra­hedra and NH_4_^+^ cations are arranged in layers perpendicular to *c* (Fig. 4 ▸). The NH_4_^+^ cation is ten-coordinated, with an average N—O bond distance of 3.112 Å (Table 4 ▸), leading to a BVS of 0.97 v.u. (García-Rodríguez *et al.*, 2000 ▸). Both AsO_4_ groups are overbonded (5.08 and 5.13 v.u. for As1 and As2, respectively), although the average As—O bond lengths (1.682 and 1.678 Å) are fairly close to the statistical average of 1.687 Å (Gagné & Hawthorne, 2018*a* ▸). All O atoms are con­siderably underbonded and participate in a complex hydro­gen-bonding network (Table 5 ▸). In Rb(H_2_AsO_4_)(H_3_AsO_4_) (Naili & Mhiri, 2001 ▸), there are some very strong hydrogen bonds present (2.432 Å) that connect the structure along the *c* axis. Hydrogen bonds with O—H⋯O distances < 2.5 Å are also present in many isostoichiometric *M* ^+^H_5_(PO_4_)_2_ com­pounds [see compilation in Naili & Mhiri (2001 ▸)]. In (NH_4_)(H_2_AsO_4_)(H_3_AsO_4_), these O—H⋯O hydrogen bonds are still strong but considerably longer, ranging from 2.568 (8) to 2.653 (9) Å. This is probably due to a small shift of the atom positions in the two compounds, seen also from an inspection of the unit cells of the two homeotypic compounds. While unit-cell parameters *a* and *b* are quite similar and 0.003 and 0.033 Å longer, respectively, in the ammonium compound, unit-cell parameter *c* is considerably shorter [19.623 (4) Å; Table 1 ▸] in comparison with that of the rubidium compound [20.4226 (6) Å; Naili & Mhiri, 2001 ▸], leading also to a distinctly smaller unit-cell volume of (NH_4_)(H_2_AsO_4_)(H_3_AsO_4_). This change is explained, unlike what is expected from the slightly different effective ionic radii of NH_4_^+^ and Rb^+^ (the latter is slightly smaller), firstly, by the ability of the NH_4_^+^ cation to form hydrogen bonds, and, secondly, by a slight shift of the As1 atoms in the *b* direction and a slight expansion in that direction. Hydrogen bonds connecting adjacent As2O_4_ tetra­hedra in the *b* direction in Rb(H_2_AsO_4_)(H_3_AsO_4_) are lost and replaced by hydrogen bonds connecting As1O_4_ and As2O_4_ along *c* in (NH_4_)(H_2_AsO_4_)(H_3_AsO_4_) (Fig. 5 ▸), resulting in a compression of the whole structure along *c*.

**Figure 6:**
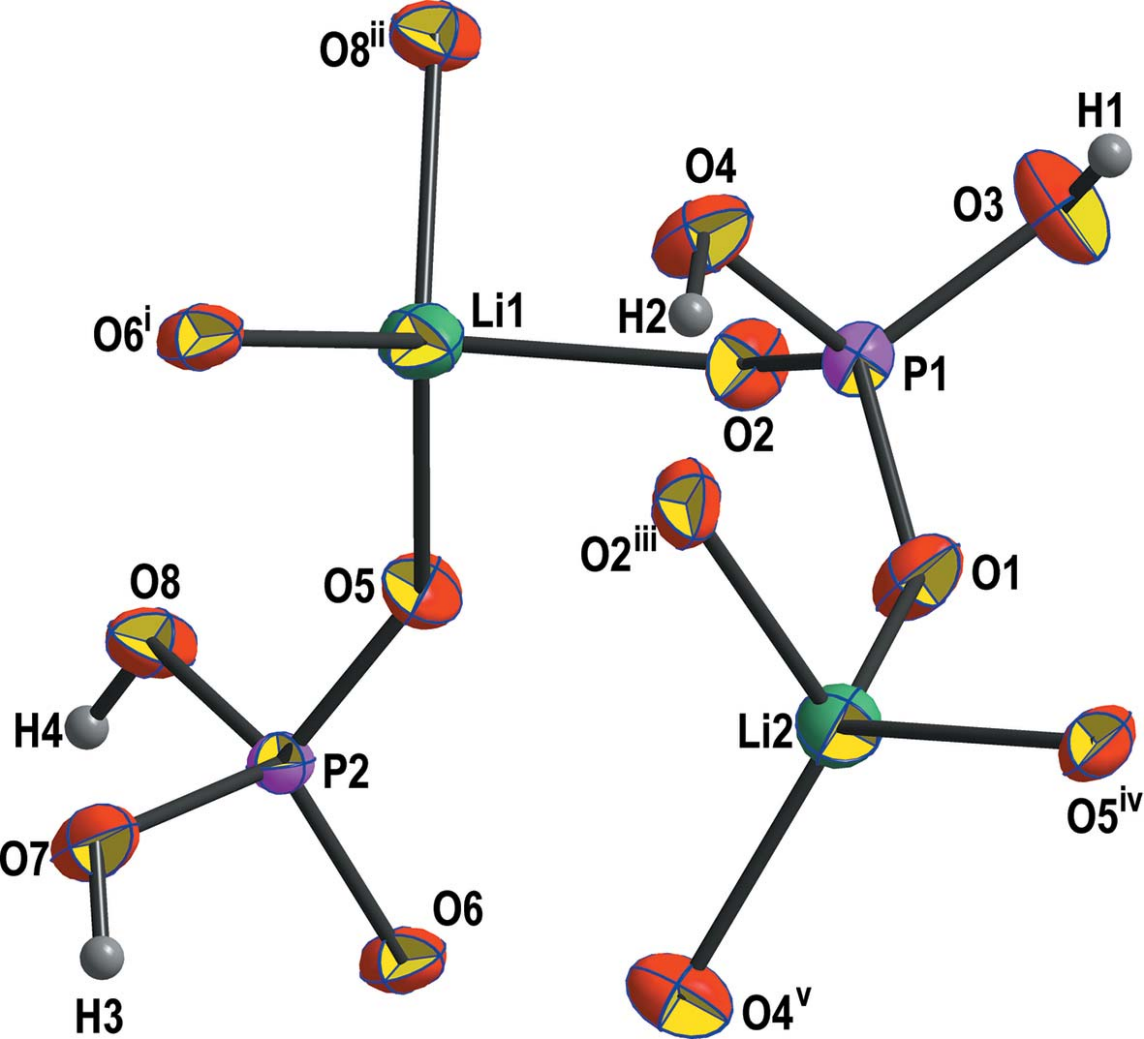
The principal building unit of Li_2_(H_2_PO_4_)_2_, shown with displacement ellipsoids at the 70% probability level. The symmetry codes are as defined in Table 7 ▸.

**Figure 7:**
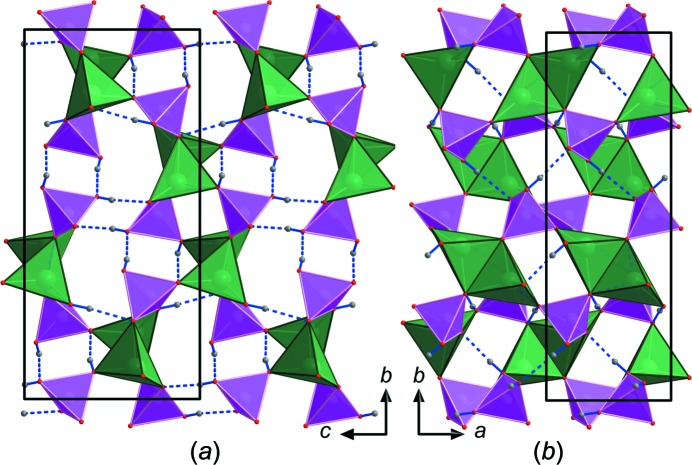
Structural drawings of Li_2_(H_2_PO_4_)_2_, viewed along (*a*) *a* and (*b*) *c*. The unit cell is outlined. Phosphate tetra­hedra are shown in pink and edge-sharing LiO_4_ tetra­hedra in green. The hydrogen bonds reinforcing the network are shown in blue.

The asymmetric unit of monoclinic (*P*2_1_/*n*) Li_2_(H_2_PO_4_)_2_ contains two Li, two P, eight O and four H atoms, all in general positions (Fig. 6 ▸). Li_2_(H_2_PO_4_)_2_ is built of LiO_4_ tetra­hedra that share edges with adjacent LiO_4_ tetra­hedra, thereby forming Li_2_O_6_ dimers (Fig. 7 ▸ *b*). Each corner of the LiO_4_ tetra­hedra shares a corner with a PO_4_ tetra­hedron, thus connecting the Li_2_O_6_ dimers into a 3D network (Figs. 7 ▸ *a* and 7*b*). This network is reinforced by hydrogen bonds of medium-to-high strength (Table 6 ▸). In the ortho­rhom­bic (*Pna*2_1_) dimorph of Li(H_2_PO_4_) (Catti & Ivaldi, 1978 ▸), which is characterized by a high electrical (proton) conductivity (Hwan Oh *et al.*, 2010 ▸), the LiO_4_ tetra­hedra share corners, thus forming chains that are connected by the PO_4_ groups. In monoclinic Li_2_(H_2_PO_4_)_2_, the average (Table 7 ▸) Li—O (1.951 and 1.953 Å) and P—O (1.539 and 1.537 Å) bond lengths are very close to the statistical average of 1.972 Å (Gagné & Hawthorne, 2016 ▸) for Li—O and 1.537 Å (Gagné & Hawthorne, 2018*b* ▸) for P—O bond lengths. This is also reflected by the nearly ideal BVSs (Gagné & Hawthorne, 2015 ▸) of 1.01 and 1.00 v.u. for Li1 and Li2, respectively, and 4.98 and 5.00 v.u. for P1 and P2, respectively. The most underbonded O atoms (O3, O4, O7 and O8, with BVSs of 1.16–1.37 v.u.) form strong-to-medium hydrogen bonds (Table 6 ▸). A comparison of the X-ray densities of monoclinic Li_2_(H_2_PO_4_)_2_ (2.123 kg m^−3^) and its ortho­rhom­bic dimorph LiH_2_PO_4_ (Catti & Ivaldi, 1978 ▸) (2.09 kg m^−3^) suggests that monoclinic Li_2_(H_2_PO_4_)_2_ is slightly denser and therefore thermodynamically slightly more stable, at least under ambient conditions. Ortho­rhom­bic LiH_2_PO_4_ shows no phase transition between room temperature and 100 (Hwan Oh *et al.*, 2010 ▸) or 17 K (Lee *et al.*, 2008 ▸). We note that monoclinic Li_2_(H_2_PO_4_)_2_ most probably has an isotypic arsenate analogue, since Remy & Bachet (1967 ▸) were able to synthesize monoclinic Li_2_(H_2_AsO_4_)_2_, with *a* = 5.55, *b* = 16.36, *c* = 7.80 Å, β = 90.53° and space group *P*2_1_/*n*, although they did not determine its crystal structure. Ortho­rhom­bic Li(H_2_PO_4_) also has an isotypic arsenate analogue, the crystal structure of which was reported by Fanchon *et al.* (1987 ▸), who pointed out a slight rearrangement in one of the two independent hydrogen bonds.

## Statistical evaluation of As—O bonds in protonated AsO_4_ groups   

**Figure 8:**
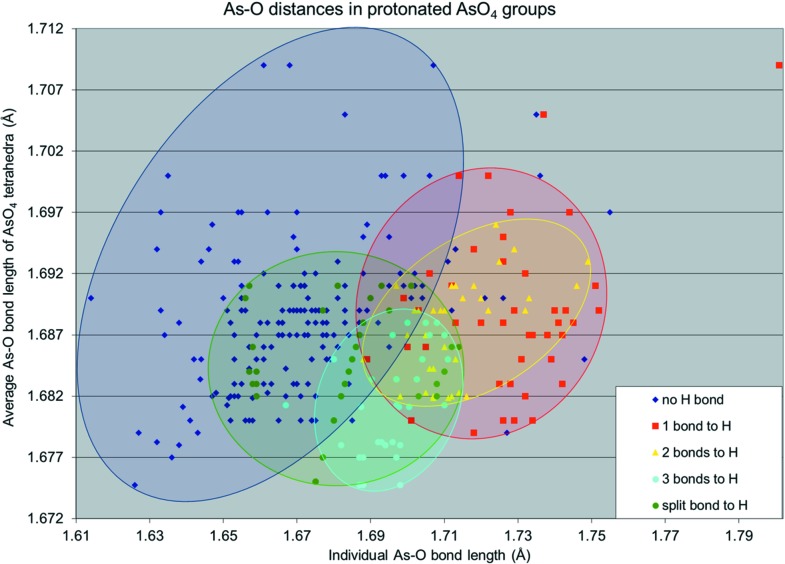
Comparison of As—O distances in H_*n*_AsO_4_ (*n* = 1–3) groups, sorted by As—OH bonds into clouds for H_3_AsO_4_ (turquoise), H_2_AsO_4_ (yellow) and HAsO_4_ (red) groups. As—OH bonds to split H-atom positions are shown in green, while all bonds to the remaining nonprotonated O atoms are shown in blue.

Several statistical analyses of bond lengths in As^5+^O_4_ polyhedra have been published recently. Gagné & Hawthorne (2018*a* ▸) reported average As—O bond lengths of 1.687 (27) Å in AsO_4_ and 1.830 (28) Å in AsO_6_ groups, derived from 508 and 13 polyhedra, respectively. Schwendtner (2008 ▸) found similar values of 1.686 (29) and 1.827 (29) Å for a larger sample size of 704 AsO_4_ and 40 AsO_6_ polyhedra, respectively. An analysis of As—O bond lengths in minerals by Majzlan *et al.* (2014 ▸) gave a very similar value of 1.685 Å (no s.u. given) for the average As—O bond length and a value of 1.727 Å (no s.u. given) for As—OH bonds. Data for As—O bond lengths in multiply protonated As^5+^O_*x*_ (*x* = 4 and 6) polyhedra are scarce (especially those for H_3_AsO_4_ groups) due to the rare occurrence of compounds containing such polyhedra. An earlier attempt by Ichikawa (1988 ▸) to carry out a statistical analysis of the hydrogen-bond-length dependence of the distortion in H_*n*_AsO_4_ (*n* = 1–3) tetra­hedra was severely hampered for the doubly and triply protonated representatives, since data for only six H_2_AsO_4_ and two H_3_AsO_4_ groups were available, and no pertinent conclusions were possible. As the number of synthetic compounds and minerals containing H_*n*_AsO_4_ (*n* = 1–3) groups has considerably increased in the last three decades, we were able to perform a detailed analysis of As—O/OH bonds in H_*n*_AsO_4_ (*n* = 1–3) groups using data from the ICSD database (FIZ, 2018 ▸) (conventional *R* value < 5, full occupancy of As and O sites), expanded by the published data for known H_3_AsO_4_-containing inorganic compounds men­tioned in the *Introduction* (§1), and the two novel title arsenate compounds (Table 8 ▸ and Fig. 8 ▸).

The average As—O/OH bond length for the 97 analysed H_*n*_AsO_4_ (*n* = 1–3) groups of 1.686 (27) Å is nearly identical to the value reported by Gagné & Hawthorne (2018*a* ▸), but the individual bond lengths vary greatly with the number of As—OH bonds in the respective polyhedra. While the As—OH bonds are extremely elongated to 1.728 (19) Å in HAsO_4_ groups and to 1.714 (12) Å in H_2_AsO_4_ groups, the average As—OH bond length is considerably shorter, with a value of 1.694 (16) Å in the rare H_3_AsO_4_ groups. This result is in agreement with the observation of Ferraris & Ivaldi (1984 ▸) that the average length of *X*—OH (*X* = As and P) bonds tends to decrease from mono- to triprotonated anions with the same *X* atom. We also find that the As bonds to nonprotonated O atoms in H_3_AsO_4_ groups are shortened to 1.671 (23) Å. If As—O bonds involving bridging O ligands (as present in the As_2_O_7_ groups in pyroarsenates), *i.e.* As—O—As bonds, are removed from the data set because they are known to be anomalously elongated due to As—As repulsion, the value is even shorter, *i.e.* 1.667 (18) Å. A special case are As—O bonds to half-occupied H-atom positions; these are actually shortened to 1.683 (13) Å. Excluding split H-atom positions, the grand mean average As—OH bond length in H_*n*_AsO_4_ (*n* = 1–3) groups is 1.714 (21) Å and thus considerably shorter than the value of 1.727 Å derived by Majzlan *et al.* (2014 ▸), whose evaluation was based mainly on H_1-2_AsO_4_ groups. A visual analysis of the individual As—O bond lengths compared to the averages of the H_*n*_AsO_4_ (*n* = 1–3) groups (Fig. 8 ▸) shows that they form clearly distributed clouds, depending on the number of H atoms. The average As—O/OH bond lengths of the polyhedra, as well as the individual As—OH bond lengths, are largest in HAsO_4_ groups and show a narrower distribution in H_2_AsO_4_. The population of H_3_AsO_4_ groups is characterized by shorter individual As—OH bond lengths but also a shorter average As—OH bond length of the polyhedra. It can also be recognized that the whole data set shows a strong concentration of bonds at around *ca* 1.687 Å and that all the shortest bonds are to the nonprotonated O atoms of each H_*n*_AsO_4_ (*n* = 1–3) group (blue cloud in Fig. 8 ▸, *cf.* Table 8 ▸). This is expected because the As atom in each H_*n*_AsO_4_ tries to achieve a BVS of 5, and due to the elongation of all the bonds to protonated O atoms, the remaining As—O bonds have to shorten accordingly. This also explains why both the individual As—OH bond lengths and average As—O(H) bond lengths decrease with increasing protonation. In the case of singly protonated AsO_4_ groups, the three As—O bonds need to become slightly shortened in order to still achieve a BVS of 5, at the expense of a high bond-length distortion in this tetra­hedron. In agreement with the distortion theorem (Brown & Shannon, 1973 ▸), this results in a slightly higher value of the average As—O(H) bond length of 1.689 (6) Å in HAsO_4_ groups (vertical range of red cloud in Fig. 8 ▸) *versus* a corresponding value of 1.688 (3) Å in H_2_AsO_4_ groups (vertical range of yellow cloud) and the notably lower value of 1.680 (7) Å in H_3_AsO_4_ groups (vertical range of turquoise cloud). This low value in the latter is a consequence of three competing As—OH bonds which can only be counteracted by one As—O bond. This leads to three similarly short As—OH bonds and one even shorter As—O bond, *i.e.* a small bond-length distortion.

The overall spread of values is a consequence of the variable strengths of the hydrogen bonds in the individual compounds. A conspicuous outlier in Fig. 8 ▸ (*e.g.* in the top-right corner) may be explained by the influence of a very strong hydrogen bond in Mg(HAsO_4_)(H_2_O)_7_, with an O⋯O donor–acceptor distance of 2.491 Å (no s.u. given; Ferraris & Franchini-Angela, 1973 ▸).

## Supplementary Material

Crystal structure: contains datablock(s) CsH2AsO4H3AsO42, Li2H2PO42, NH4H2AsO4H3AsO4, global. DOI: 10.1107/S2053229619008489/qf3024sup1.cif

Structure factors: contains datablock(s) CsH2AsO4H3AsO42. DOI: 10.1107/S2053229619008489/qf3024CsH2AsO4H3AsO42sup2.hkl

Structure factors: contains datablock(s) Li2H2PO42. DOI: 10.1107/S2053229619008489/qf3024Li2H2PO42sup3.hkl

Structure factors: contains datablock(s) NH4H2AsO4H3AsO4. DOI: 10.1107/S2053229619008489/qf3024NH4H2AsO4H3AsO4sup4.hkl

Supporting information file. DOI: 10.1107/S2053229619008489/qf3024Li2H2PO42sup5.cml

Supporting information file. DOI: 10.1107/S2053229619008489/qf3024NH4H2AsO4H3AsO4sup6.cml

CCDC references: 1922991, 1922990, 1922989

## Figures and Tables

**Table 1:** Experimental details Experiments were carried out at 293 K with Mo *K*α radiation using a Nonius KappaCCD single-crystal four-circle diffractometer. Absorption was corrected for by multi-scan methods (*SCALEPACK*; Otwinowski *et al.*, 2003 ▸).

	**Cs(H_2_AsO_4_)(H_3_AsO_4_)_2_**	**(NH_4_)(H_2_AsO_4_)(H_3_AsO_4_)**	**Li_2_(H_2_PO_4_)_2_**
Crystal data			
Chemical formula	Cs(H_2_AsO_4_)(H_3_AsO_4_)_2_	(NH_4_)(H_2_AsO_4_)(H_3_AsO_4_)	Li_2_(H_2_PO_4_)_2_
*M*_r_	557.73	300.92	207.85
Crystal system, space group	Monoclinic, *P*2_1_/*c*	Orthorhombic, *P* *b* *c* *a*	Monoclinic, *P*2_1_/*n*
*a*, *b*, *c* (Å)	9.712 (2), 12.738 (3), 9.307 (2)	7.943 (2), 9.855 (2), 19.623 (4)	5.400 (1), 15.927 (3), 7.562 (2)
α, β, γ (°)	90, 90.91 (3), 90	90, 90, 90	90, 90.47 (3), 90
*V* (Å^3^)	1151.2 (4)	1536.1 (6)	650.4 (2)
*Z*	4	8	4
μ (mm^−1^)	11.83	8.71	0.67
Crystal size (mm)	0.14 × 0.13 × 0.08	0.15 × 0.10 × 0.07	0.15 × 0.12 × 0.10

Data collection			
*T*_min_, *T*_max_	0.288, 0.451	0.355, 0.581	0.906, 0.936
No. of measured, independent and observed [*I* > 2σ(*I*)] reflections	8200, 4186, 3411	1799, 1295, 905	5625, 2857, 2490
Completeness to 0.84 Å resolution	1.00	0.65	1.00
*R*_int_	0.016	0.038	0.014
(sin θ/λ)_max_ (Å^−1^)	0.758	0.676	0.806

Refinement			
*R*[*F* ^2^ > 2σ(*F* ^2^)], *wR*(*F* ^2^), *S*	0.023, 0.054, 1.05	0.046, 0.109, 1.02	0.025, 0.072, 1.04
No. of reflections	4186	1295	2857
No. of parameters	178	136	126
No. of restraints	1	9	0
H-atom treatment	All H-atom parameters refined	Only H-atom coordinates refined	All H-atom parameters refined
Δρ_max_, Δρ_min_ (e Å^−3^)	0.91, −1.60	0.74, −0.61	0.44, −0.38

Computer programs: *COLLECT* (Nonius, 2003 ▸), *DENZO* and *SCALEPACK* (Otwinowski *et al.*, 2003 ▸), *SHELXS97* (Sheldrick, 2008 ▸), *SHELXL2016* (Sheldrick, 2015 ▸), *DIAMOND* (Brandenburg, 2005 ▸) and *publCIF* (Westrip, 2010 ▸).

**Table 2:** Selected bond lengths (Å) for Cs(H_2_AsO_4_)(H_3_AsO_4_)_2_

Cs1—O6^i^	3.1202 (17)	As1—O1	1.6437 (15)
Cs1—O2^ii^	3.2184 (19)	As1—O2	1.6903 (17)
Cs1—O3^iii^	3.2326 (19)	As1—O3	1.6970 (17)
Cs1—O4	3.2469 (17)	As1—O4	1.7025 (16)
Cs1—O5^iv^	3.2536 (18)	As2—O5	1.6390 (16)
Cs1—O1^v^	3.3579 (17)	As2—O6	1.6874 (16)
Cs1—O11^vi^	3.359 (2)	As2—O7	1.6977 (19)
Cs1—O12	3.478 (2)	As2—O8	1.7004 (19)
Cs1—O9^vii^	3.7056 (19)	As3—O9	1.6515 (16)
Cs1—O11^viii^	3.755 (3)	As3—O10	1.6579 (17)
Cs1—O8	3.844 (2)	As3—O12	1.707 (2)
Cs1—O7^vii^	3.924 (3)	As3—O11	1.7104 (19)

Symmetry codes: (i) 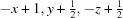; (ii) 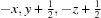; (iii) 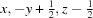; (iv) 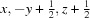; (v) 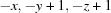; (vi) 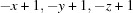; (vii) 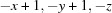; (viii) 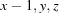.

**Table 3:** Hydrogen-bond geometry (Å, °) for Cs(H_2_AsO_4_)(H_3_AsO_4_)_2_

*D*—H⋯*A*	*D*—H	H⋯*A*	*D*⋯*A*	*D*—H⋯*A*
O2—H2⋯O10^viii^	0.83 (4)	1.70 (4)	2.524 (2)	171 (4)
O3—H3⋯O9^ix^	0.79 (4)	1.76 (4)	2.553 (3)	172 (4)
O4—H4⋯O1^iii^	0.92 (3)	1.70 (3)	2.609 (2)	170 (3)
O6—H6⋯O10	0.91 (2)	1.64 (2)	2.539 (2)	170 (4)
O7—H7⋯O9^vii^	0.81 (4)	1.79 (4)	2.599 (3)	177 (4)
O11—H11⋯O1^vi^	0.79 (4)	1.85 (4)	2.630 (3)	168 (4)
O8—H8⋯O5^iv^	0.82 (4)	1.85 (4)	2.664 (2)	170 (4)
O12—H12⋯O5^iv^	0.81 (4)	1.84 (4)	2.643 (3)	171 (4)

Symmetry codes: (iii) 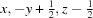; (iv) 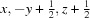; (vi) 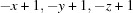; (vii) 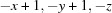; (viii) 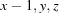; (ix) 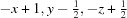.

**Table 4:** Selected bond lengths (Å) for (NH_4_)(H_2_AsO_4_)(H_3_AsO_4_)

N—O5	2.869 (10)	N—O3^i^	3.283 (10)
N—O1^i^	2.947 (9)	As1—O1	1.648 (5)
N—O5^i^	3.032 (11)	As1—O2	1.662 (6)
N—O2^ii^	3.075 (9)	As1—O3	1.705 (6)
N—O4^iii^	3.082 (9)	As1—O4	1.714 (5)
N—O6	3.148 (10)	As2—O5	1.632 (6)
N—O7^iv^	3.194 (10)	As2—O8	1.692 (5)
N—O3	3.216 (10)	As2—O7	1.693 (5)
N—O8^v^	3.272 (9)	As2—O6	1.696 (6)

Symmetry codes: (i) 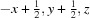; (ii) 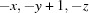; (iii) 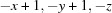; (iv) 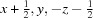; (v) 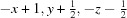.

**Table 5:** Hydrogen-bond geometry (Å, °) for (NH_4_)(H_2_AsO_4_)(H_3_AsO_4_)

*D*—H⋯*A*	*D*—H	H⋯*A*	*D*⋯*A*	*D*—H⋯*A*
N—H1⋯O4^iii^	0.89 (2)	2.36 (8)	3.082 (9)	138 (10)
N—H1⋯O7^iv^	0.89 (2)	2.54 (9)	3.194 (10)	130 (9)
N—H4⋯O5^i^	0.90 (2)	2.20 (7)	3.032 (11)	154 (14)
N—H3⋯O1^i^	0.89 (2)	2.10 (4)	2.947 (9)	159 (9)
N—H2⋯O5	0.91 (2)	1.96 (2)	2.869 (10)	174 (7)
O3—H5⋯O1^vi^	0.89 (2)	2.13 (15)	2.616 (7)	113 (12)
O3—H5⋯O3^ii^	0.89 (2)	2.61 (11)	3.311 (12)	136 (13)
O6—H7⋯O2^vii^	0.90 (2)	1.82 (10)	2.653 (9)	152 (19)
O4—H6⋯O1^i^	0.89 (2)	1.77 (3)	2.650 (8)	170 (7)
O7—H8⋯O2^viii^	0.89 (2)	1.72 (4)	2.568 (8)	157 (8)
O8—H9⋯O5^iv^	0.89 (2)	1.78 (6)	2.590 (7)	150 (11)

Symmetry codes: (i) 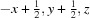; (ii) 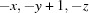; (iii) 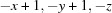; (iv) 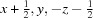; (vi) 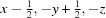; (vii) 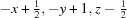; (viii) 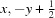, 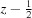.

**Table 6:** Hydrogen-bond geometry (Å, °) for Li_2_(H_2_PO_4_)_2_

*D*—H⋯*A*	*D*—H	H⋯*A*	*D*⋯*A*	*D*—H⋯*A*
O3—H1⋯O7^vi^	0.77 (2)	1.91 (2)	2.6769 (12)	171 (2)
O4—H2⋯O2^iv^	0.84 (2)	1.99 (2)	2.8292 (14)	176 (2)
O7—H3⋯O6^vii^	0.73 (2)	1.79 (2)	2.5210 (13)	172 (3)
O8—H4⋯O1^viii^	0.79 (2)	1.79 (2)	2.5667 (12)	167 (2)

Symmetry codes: (iv) 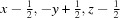; (vi) 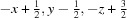; (vii) 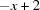, 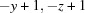; (viii) 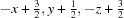.

**Table 7:** Selected bond lengths (Å) for Li_2_(H_2_PO_4_)_2_

Li1—O5	1.888 (2)	Li2—P2^iv^	3.077 (2)
Li1—O6^i^	1.902 (2)	P1—O1	1.4996 (9)
Li1—O8^ii^	1.967 (2)	P1—O2	1.5043 (8)
Li1—O2	2.045 (2)	P1—O3	1.5588 (9)
Li1—Li2^iii^	2.611 (3)	P1—O4	1.5917 (8)
Li1—P2^i^	3.068 (2)	P2—O5	1.4944 (8)
Li2—O5^iv^	1.919 (2)	P2—O6	1.5113 (8)
Li2—O1	1.944 (2)	P2—O7	1.5640 (9)
Li2—O4^v^	1.973 (2)	P2—O8	1.5774 (8)
Li2—O2^iv^	1.974 (2)		

Symmetry codes: (i) 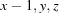; (ii) 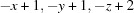; (iii) 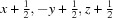; (iv) 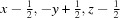; (v) 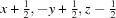.

**Table 8:** Statistical analysis of the As—O bond lengths (Å) in H_*n*_AsO_4_ (*n* = 1–3) groups

Bond lengths	Analysed number	Average	Minimum	Maximum
As—O/OH in H_*n*_AsO_4_ (average)	97	1.687 (6)	1.660	1.709
As—O/OH in H_*n*_AsO_4_ (individual)	388	1.687 (27)	1.614	1.801
As—OH in H_*n*_AsO_4_ (including split H-atom positions)	199	1.701 (23)	1.625	1.801
As—OH in H_*n*_AsO_4_ (no split H atoms)	117	1.714 (21)	1.625	1.801
As—OH in HAsO_4_	43	1.728 (19)	1.689	1.801
As—OH in H_2_AsO_4_	41	1.714 (12)	1.688	1.749
As—OH in H_3_AsO_4_	33	1.694 (16)	1.625	1.712
As—OH/2 (split H atoms) in H_1–2_AsO_4_	82	1.683 (13)	1.656	1.714
As—O (no H atoms) in H_*n*_AsO_4_	189	1.671 (23)	1.614	1.755
As—O (no H/As*) in H_*n*_AsO_4_	174	1.667 (18)	1.614	1.735

Note: (*) no As—O—As bonds (see text).
